# Cytoplasmic Beta and Gamma Actin Isoforms Reorganization and Regulation in Tumor Cells in Culture and Tissue

**DOI:** 10.3389/fphar.2022.895703

**Published:** 2022-05-26

**Authors:** V. B. Dugina, G. S. Shagieva, P. B. Kopnin

**Affiliations:** ^1^ A.N. Belozerskiy Research Institute of Physico-Chemical Biology, M.V. Lomonosov Moscow State University, Moscow, Russia; ^2^ Biological Faculty, M.V. Lomonosov Moscow State University, Moscow, Russia; ^3^ Research Institute of Carcinogenesis, N.N. Blokhin Russian Cancer Research Center, Ministry of Health of Russia, Moscow, Russia

**Keywords:** cytoplasmic actin isoforms, beta actin, gamma actin, neoplastic transformation, cytoskeleton

## Abstract

The cytoplasmic actin isoforms (β- and γ-actins) contribute greatly to cellular processes such as cel-cell and cell-matrix interactions, as well as cell polarization, motility and division. Distinct isoforms modulations are linked to serious pathologies, so investigations of underlying mechanisms would be of major relevance not only for fundamental research but also for clinical applications. Therefore, the study of the relevant mechanisms of change in the isoform’s balance is important for basic research and for clinical studies. The disruption of actin cytoskeleton and intercellular adhesions contribute to the neoplastic transformation, as it is important for the tumor growth, invasiveness and metastasis. Cytoplasmic actins display the functional diversity: β-actin is responsible for contractility, whereas γ-actin participates in the submembrane flexible cortex organization and direction cell motility. The involvement of β- and γ-actin in cell architecture, motility, division, and adhesion junctions in normal cells is not equivalent, and the major question was following: whether isoform ratio and the distribution in the cell corresponds to pathological function. Significant data were obtained in the study of tumor and normal cells in culture, as well as on clinical material of human tissues, and via selective regulation of β- and γ-actin’s expression. Investigation of the actins’ diversity and function in cancers may help to choose the benefit treatment strategies, and to design new therapies.

## Actin

Eukaryotic cells contain three different cytoskeletal systems: actin microfilaments, microtubules, and intermediate filaments. They have different architectural organization, dynamic and mechanical properties. Actin is one of the major proteins and is highly conserved between the species.

Actin is responsible for the performance of mechanical functions, providing the maintenance of the cell shape and motility, contractility, tension, cell division, and nuclear functions. The actin system reorganization in the cell is feasible owing to the reversible transition from a monomeric to a polymeric form of actin, as well as to the interaction of this protein with a variety of actin binding proteins (ABPs), regulating the architecture of actin filament system in the cell. Microfilament disorganization leads to the changes in cell morphology, proliferation, and to aberrant response to the extracellular signaling.

## Actin Isoforms

Six different actin isoforms are expressed in a higher mammal (Vandekerckhove and Weber, 1978). The primary structure of actin is very conservative from birds to humans (Kabsch and Vandekerckhove, 1992). Actin isoforms are coded for by a set of structurally related genes that originate from a common precursor and have highly homologous nucleotide sequences (Hightower and Meagher, 1986). The main differences between isoforms are concentrated at the N-terminus (Vandekerckhove and Weber, 1978). Six human actin genes - α-skeletal (*ACTA1*), α-cardiac (*ACTC1*), α-smooth muscle (*ACTA2*), γ-smooth muscle (*ACTG2*), β-cytoplasmic (*ACTB*), γ-cytoplasmic (*ACTG1*) - are localized on the different chromosomes (Gunning et al., 1984). Recently it has been declared the existence of the seventh actin isoform - β-actin-like protein 2 (*ACTBL2*), which belongs to the non-muscle actin class, like β-cytoplasmic and γ-cytoplasmic actins, but its expression is very low (Malek et al., 2021). Cytoplasmic actins are expressed in mammalian cells in various proportions and differ by four amino acid residues localized at positions 1, 2, 3, and 9 of the N-terminus (Ampe and Van Troys, 2017). The evolutionary conservatism of actins indicates their fundamental role for the cell survival.

Our review focuses on β-cytoplasmic and γ-cytoplasmic actins (β- and γ-actins hereinafter), which are expressed in the muscle and non-muscle cells in various ratios, regulated in the time and space (Garrels and Gibson, 1976; Otey et al., 1987). The actin microfilaments are organized into variety of intracellular compositions, such as microfilament bundles, lamellipodia, or filopodia. The diversity of actin structures is feasible partly due to selective interaction of actin with ABPs (Manstein and Mulvihill, 2016; Boiero Sanders et al., 2020). It is assumed that the presence of ABPs leads to the formation of isoform-specific structures within the certain cytoplasmic domains containing different actin isoforms. Two main types of actins’ organization are branched and linear networks, which are provided by the association with Arp2/3 and formins, respectively. The affinity of actin isoforms to ABPs is variable. γ-Actin directly interacts with Arp2/3 complex and WAVE2 at the leading edge during cell spreading and migration (Dugina et al., 2015). Cofilin1 which is a regulator of Arp2/3-dependent actin branching also co-localizes with γ-actin (Dugina et al., 2015). The preferential binding of γ-actin to annexin V was found (Tzima et al., 2000). Actin nucleator formin DIAPH3 selectively interacts with β-actin compared with γ-actin, which allows us to assume that filament structures constructed from DIAPH3 could be enriched with β-actin (Chen et al., 2017). Most linear but not branched actin cellular structures are interacting with particular families of ABPs-tropomyosins (Boiero Sanders et al., 2020). Linear actin networks can form structures, where all the actin filaments have the same orientation (filopodia for example) and with actin filaments of opposite polarity. The last ones in cooperation with myosin provide contractile activity of the cells. Actin structures with co-directed filaments, such as filopodia, are not contractile structures, and molecular motors are generally used for trafficking there (Boiero Sanders et al., 2020).

Therefore, strongly similar actin isoforms may be involved in the formation of various cellular structures, showing preferential binding to various ABPs.

### Functions of Actins

The three-dimensional segregation of β- and γ-actins in mesenchymal and epithelial cells during different conditions (steady state, movement, mitosis and cytokinesis) was demonstrated (Dugina et al., 2009; Dugina et al., 2018; Dugina et al., 2019; Baranwal et al., 2012; Shagieva et al., 2012; Shagieva et al., 2020). Mesenchymal cells *in vitro* express α-smooth muscle actin in addition to cytoplasmic actins (Dugina et al., 2009). β-Actin predominantly forms stress fibrils and therefore provides the tension and contractility, division and motility, maintains the cell shape. Laser confocal microscopy analysis showed that β-actin is predominantly localized in filopodia, stress fibrils, circular bundles, adhesion and focal junctions, and in the contractile ring during cytokinesis. RNA interference (Dugina et al., 2009; Shum et al., 2011; Dong et al., 2018) and CRISPR/Cas9 knockout (Malek et al., 2020) experiments revealed that γ-actin is essential for the cell shape and motility. γ-Actin is localized in a subtle system of microfilaments apically in a resting/polarized epithelial cell, in the cortical and lamellipodial networks during the cell motility, and in the submembrane domain in the mitosis (Dugina et al., 2009; Shagieva et al., 2020). Both isoforms are localized apically in the intercellular contacts in the polarized epithelial cells (Dugina et al., 2009; Baranwal et al., 2012), but β-actin is associated with adhesion junctions, and γ-actin with tight junctions (Baranwal et al., 2012).

The motility of normal fibroblasts and fibroblasts lacking β-actin or γ-actin is different (Dugina et al., 2009), so the roles of actin in cell motility are isoform-specific. It should be noted that β- and γ-actins are co-localized in the lamellipodia, both isoforms are present in the lamella, but in the different structures. β-Actin forms the bundles of microfilaments which are connected to focal adhesions, whereas γ-actin is localized in a dense network (Dugina et al., 2009). In normal fibroblasts, β-actin mRNA is concentrated on the edge of the lamella, but newly synthesized actin contributes insignificantly to the monomeric actin at the leading edge of the cell (Condeelis and Singer, 2005). The single β-actin mRNAs’ tracking has shown that the transport of these mRNAs to the focal adhesions leads to the stabilization of contacts (Katz et al., 2012).

### Actin Isoforms in the Malignant Tissues

Investigations of the specific functions of actin isoforms can be useful in the diagnosis and clarification of diverse pathologies such as fibrosis, cardiovascular and oncological diseases (Chaponnier and Gabbiani, 2004). Complex actin system reorganized at the tumor development and progression, promoting cell mobility for invasion and metastasis.

**FIGURE 1:**
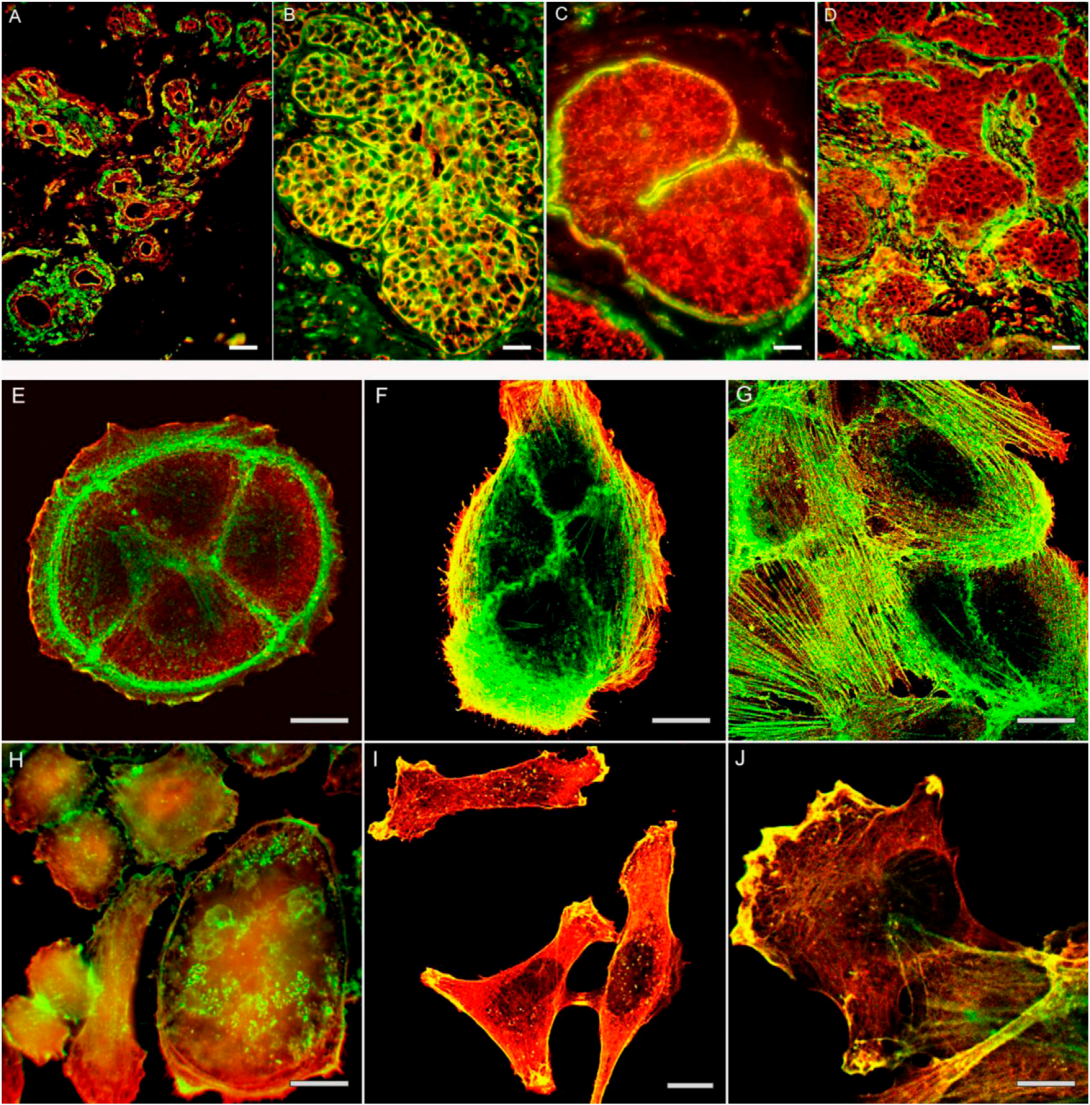
The actin cytoskeleton organization in normal mammary gland **(A)**, non-malignant proliferates **(B)** and mammary gland cancer tissue **(C,D)**. Intensive staining for β-actin in cells of proliferate **(B)**. Decreased staining for β-actin in cells of *in situ* **(C)** and invasive **(D)** cancer cells on the background of positively stained myoepithelial cells **(C)** or stromal myofibroblasts **(D)**. Bars, 50 μm. The actin cytoskeleton organization in normal epithelial cells **(E,F,G)** and cancer cells **(H,I,J)** in culture. Cytoplasmic β-actin (green) and γ-actin (red) in non-malignant HaCaT **(E)**, MCF-10 **(F,G)** epithelial cells, and cancer cell cultures SiHa (H), MDA-MB-231 **(I, J)**. Bars, 10 μm.

The ratio of β- and γ-actins is changed in the carcinoma cells in comparison with corresponding normal cells and is directly related to the growth of tumor xenografts (Dugina et al., 2015), (Figure 1A–D). Immuno-histological analysis of cervical tumor samples revealed a decrease of β-actin in comparison with normal exocervical tissue and intraepithelial neoplasia (Shagieva et al., 2012). Mutations of β-actin are occurred frequently in the diffuse large B-cell lymphoma, whereas γ-actin mutations were discovered in the multiple myeloma; these mutations were found in the regions important for actin polymerization or binding to myosin (Witjes et al., 2020). The mutations were detected in *ACTB* and *ACTG1* of lymphoid tumors, compared with negative result for myeloid hematological tumors. On the other hand, an increased *ACTB* expression has been reported in some tumor cells (Rubie et al., 2005; Ampe et al., 2021).

β-Actin down-regulation was observed in lung and colon cancer compared with non-malignant tissues. γ-Actin staining was twice as pronounced in cancer compared with normal tissue sections (Dugina et al., 2015). The impact of γ-actin to the tumor development was highlighted recently by the several studies, where a strong *ACTG1* expression was associated with an increase of metastatic potential and worse prognosis for hepatocellular carcinomas, colorectal, lung and cervical cancers (Liu et al., 2017; Chang et al., 2019; Wang et al., 2019; Yan et al., 2019). As bio-informatic research had shown, γ-actin was highly upregulated in gallbladder cancer and suggested to be used as biomarker for early stage of this type of cancer (Zali et al., 2019). Greater distant metastasis was observed in the colorectal cancer cases with enhanced γ-actin (Liu et al., 2017). 5-Hydroxymethylcytosine (5hmC) at the gene of *ACTG1* between other selected genes was sensitive and specific to cervical cancer malignancy (Wang et al., 2019). In uterine cancers, *ACTG1* displays high frequency of amplification and overexpression (Richter et al., 2020). A huge investigation of the database collections achieved 16 genes that related to the skin cancer development. Furthermore, *ACTG1* had an important expression in skin cancer. The authors supposed γ-actin to facilitate the cell proliferation and migration through ROCK signaling (Dong et al., 2018). The latest detailed data on the correlation of genetic alterations in actin genes such as mutations, amplifications, with changes in their expression in various types of tumor diseases were presented in the review of Ampe et al. (2021).

### Actin Isoforms in the Tumor Cells *in vitro*

**FIGURE 2:**
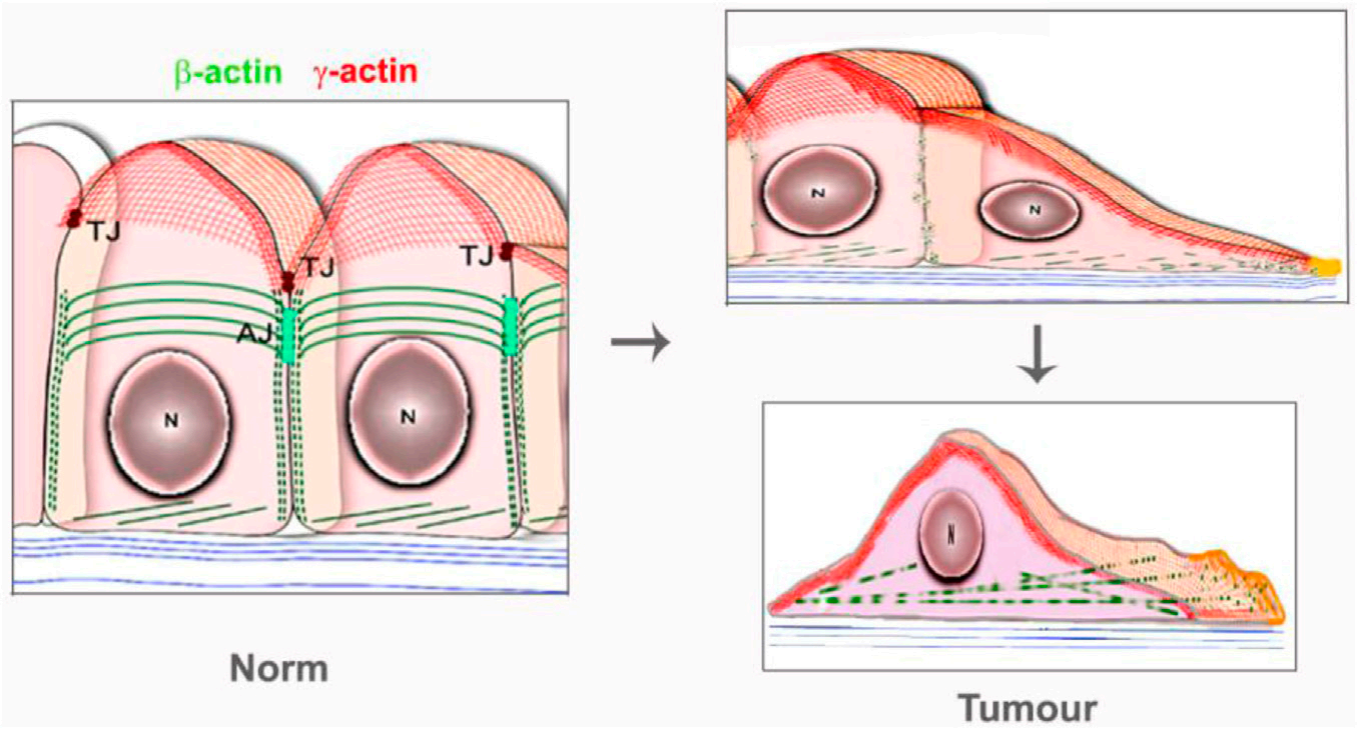
Schema of actin isoform’s systems in the normal epithelial and carcinoma cells.

The link between alterations of actins expression and tumor transformation was noted decades ago. Mutation G244D in *ACTB* caused the neoplastic transformation of human fibroblasts (Vandekerckhove et al., 1980; Leavitt et al., 1987), while mutation R28L was associated with the malignant progression of mouse melanoma cells (Sadano et al., 1988). The disappearance of actin stress fibrils in transformed cells has been demonstrated in many studies (Pollack et al., 1975; Rubin et al., 1978; Verderame et al., 1980; Pawlak and Helfman, 2001). It has been demonstrated that malignant cells contained less β-actin stress fibrils than normal counterpart, while γ-actin networks were proper developed (Dugina et al., 2008; Dugina et al., 2009; Shagieva et al., 2012), (Figure 1E–J; Figure 2, schema).

The modifications of β- or γ-actin expression caused various changes in morphology, motility, and proliferation in human cancer cells. The predominance of β-actin was in charge of a normal cell morphology, while γ-actin promoted the neoplastic features of the cells via the interconnection with the regulatory proteins ERK1/2 and PP1, as well as ABPs p34-Arc, WAVE2, and cofilin 1 (Dugina et al., 2009; Dugina et al., 2015; Dugina et al., 2016; Shum et al., 2011). Alterations of actin isoforms ratio influenced the motility of tumor cells. Partial suppression of γ-actin in neuroblastoma cells by siRNAs strongly retarded motility, provided a loss of polarity and a decrease in the velocity of the single cell migration (Shum et al., 2011). It was accompanied with an increase of the focal junctions’ number and sizes, a decrease in amount of phosphorylated paxillin, a marker of early initial contacts, and involved activation of Rho-kinase signaling pathway (Shum et al., 2011). Enhanced γ-actin promoted skin cancer cell migration via inhibition of Rho-associated kinase (ROCK) signaling pathway (Dong et al., 2018).

The impact of actin isoforms on cell cycle is divergent. The different aneuploidies and variable chromosomal rearrangements, as well as accumulation in G2/M phase were detected in mammary gland carcinoma cells after β-actin downregulation (Dugina et al., 2021). β-Actin down-regulation induced an increase of cyclins A2, B1 and D3 in breast cancer cells. On contrary, γ-actin down-regulation induced a decrease of cyclins A2, B1 and D3, and the delay in G2/M and G1 phases (Dugina et al., 2015), and γ isoform was co-localized with cyclin B1 during mitosis. Suppression of β-actin led to decrease in diploid cell population and to tetraploid cells formation (Dugina et al., 2018). γ-Actin/p-ERK1/2 co-localization was revealed in breast adenocarcinoma, lung and colon carcinoma cell lines (Dugina et al., 2015; Dugina et al., 2018). In the human hepatocellular carcinoma cells γ-actin overexpression promoted proliferation via upregulation of cyclins A2, D1, and E1 and cyclin-dependent kinases CDK2/4 and inhibited apoptosis. The knockdown of γ-actin led to the opposite effect on the cyclins and CDK’s expression, and induced G1 phase arrest (Yan et al., 2019). Reduction of γ-actin inhibited the growth of skin cancer A431 cells while the overexpression promoted the growth significantly. The clone formation and cancer cell migration were inhibited after γ-actin depletion, but enhanced by overexpression of *ACTG1* (Dong et al., 2018). The overexpression of γ-actin promoted proliferation by up-regulating cyclin-dependent kinases, inhibiting the mitochondrial apoptotic pathway, and supporting the Warburg effect, all which stimulated tumorigenicity in hepatocellular carcinomas (Yan et al., 2019). An interaction of γ-actin and glycolytic enzyme aldolase A (ALDOA) was found in metastatic lung cancer cells. ALDOA is involved in lung cancer metabolic reprogramming and metastasis, and a block of the γ-actin/ALDOA interaction by raltegravir decreased metastasis both *in vitro* and *in vivo*, and prolonged survival rate *in vivo* (Chang et al., 2019). The results suggest the benefits of compounds that block ALDOA/γ-actin interaction for patients with lung cancer and other metastatic cancers with minimal toxicity.

## Conclusion

Thereby, the cytoplasmic actins are involved in the various processes that drive neoplastic transformation, and each isoform has the unique impact to cancer development. Another isoform, α-smooth muscle actin, is already widely used in the pathological morphology. We propose that the expression of cytoplasmic actin isoforms could be used as a marker for the early cancer diagnostics, indicate the efficacy of currently used treatment, and highlight the potential targets in the tumor therapy. Further research in cancer patient genomes may provide new data needed for accurate diagnosis and effective therapy of tumor diseases with actin-related disorders (Ampe et al., 2021).
